# Metallic Coatings Boost the Cooling Power of Nanoporous Alumina

**DOI:** 10.1021/acsaenm.4c00245

**Published:** 2024-07-29

**Authors:** Alba Díaz-Lobo, Marisol Martin-Gonzalez, Qimeng Song, Ángel Morales-Sabio, Markus Retsch, Cristina V. Manzano

**Affiliations:** † Instituto de Micro y Nanotecnología, IMN-CNM, CSIC (CEI UAM + CSIC), Isaac Newton, 8, E-28706 Tres Cantos, Madrid, Spain; ‡ Department of Chemistry, Physical Chemistry I, University of Bayreuth, 95447 Bayreuth, Germany; § Centro de Investigaciones Energéticas, Medioambientales y Tecnológicas (CIEMAT), Avda. Complutense, 22, E-28040 Madrid, Spain

**Keywords:** anodic aluminum oxide (AAO), metal coating, passive radiative cooling, emissivity, thermal emitters, temperature reduction

## Abstract

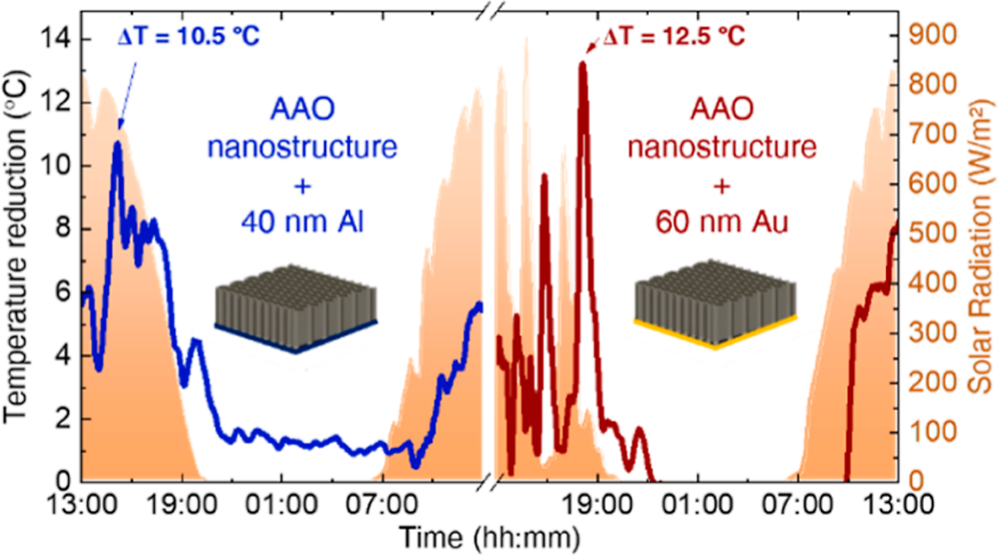

Passive daytime radiative cooling (PDRC) has emerged as a promising strategy to mitigate the increasing impact of heat waves. However, achieving effective PDRCs requires cost-effective, ecofriendly, and industrially scalable materials. In this study, we investigate the potential of anodic aluminum oxide (AAO) nanostructures coated with metals as passive radiative coolers. We explore the effects of different metallic coatings (Al and Au) with varying thicknesses (ranging from 20 to 100 nm) on the cooling performance of the AAO nanostructures. Our finding reveals a maximum temperature reduction (Δ*T*) of 12.5 °C for 60 nm of Au coating. Furthermore, we demonstrate the dependence of the cooling performance on ambient temperature, emphasizing the practical benefits of these enhanced AAO-based radiative coolers for real-world applications. Notably, our results surpass previous works, offering an avenue to enhance the PDRC capability.

## 1 Introduction

The escalating global climate crisis necessitates innovative solutions for managing heat stress. Air conditioning, the prevailing solution, is deemed unsustainable due to its high energy consumption and the subsequent expenses that it incurs. As temperatures continue to rise, relying solely on increased air conditioning consumption is not a viable path forward. Therefore, learning to manage the heat flows of everyday life in an unusual way is a major task ahead, where passive daytime radiative cooling (PDRC) offers an alternative paradigm.^1,2^ By leveraging natural heat exchange processes, the PDRC aims to dissipate excess heat from our surroundings. The concept is simple yet powerful: during scorching days, PDRC systems release heat into the vastness of outer space through atmospheric windows.^3,4^ Remarkably, this approach also adapts to milder weather conditions, maintaining thermal comfort without an active energy input. However, most PDRC promising materials require complex and expensive fabrication processes^5−9^ or vacuum systems^10^ to achieve high solar reflectivity and strong thermal emission.

Herein, a simple and low-cost method to enhance the PDRC performance of nanostructured anodic aluminum oxide (AAO) by applying metallic coatings is proposed. As with other porous materials,^11−17^ AAO has been shown to exhibit good PDRC properties^18^ due to its high porosity and emissivity, with the advantage that it can be easily fabricated by anodizing aluminum foils.^19,20^Table 1 summarizes some examples of works using alumina to develop passive radiative coolers.

**Table 1:** Summary of Previous Works Where AAO Has Been Used

materials	thickness (μm)	*R*_sol_ (%)	ε_IR_ (%)	*P*_cool_ (W/m^2^)	Δ*T* (°C)	solar intensity (W/m^2^)	*T*_amb_ (°C)	refs
AAO + AAO glue + Al	56.8	99.4	90	64	2.6		306	(21)
AAO-Al	12	89	97	175	8.0	800	320	(18)
AAO + NTs SiO_2_ + Ag + PDMS	20	95	98	71	6.7		312	(22)
AAO + SiO_2_ + Ti/Ag	50	86	96	65.6	6.1		308	(23)
PDMS + Al_2_O_3_	500	∼0.95	>0.96	∼90.8	5.1	862	305	(24)
SiO_2_/Si_3_N_4_/Al_2_O_3_/Ag	502	94.8	0.87	66.4	7.6	901	303	(25)
PVDF/Al_2_O_3_	500	97	0.95	82.7	4.0	850	312	(26)

However, AAO also has some drawbacks such as low solar reflectivity, high thermal conductivity, and poor mechanical stability. We hypothesize that coating AAO with different metals can improve its PDRC performance by increasing its solar reflectivity, reducing its thermal conductivity, and strengthening its mechanical stability.

There are very few works in which AAO nanostructures are combined with metals: Fu et al.^21^ and Diaz-Lobo et al.^18^ used an Al substrate, while Lee et al.^23^ and Zhou et al.^22^ used an Ag substrate. Researchers have tested how well AAO nanostructures cool Al and Ag substrates, but they have not investigated how the type and thickness of the metallic layer affect the cooler’s performance. This is something that needs to be thought about.

Therefore, we fabricated identical AAO nanostructures and coated them with two different metals, Al and Au. These metals have been chosen because they exhibit high reflectivity^27^ in the solar spectrum wavelengths range. Although other metals such as Ag and Cu also show high reflectivity for wavelengths longer than 1200 nm, they have not been included in this work. The selection was based on both the inertness of Au, to prevent undesirable oxidation process, and the good optical properties of aluminum oxide for passive radiative cooling applications. We also use different thicknesses for the metals (from 20 nm, where the AAO surface is not perfectly covered, to 100 nm, which covers the AAO surface perfectly and is thick enough to avoid transmission) to study the effect of the metallic coating with distinct thicknesses on the AAO nanostructures’ passive radiative cooling properties and on their optical response. The relation of these properties with the morphology of the metallic layer grown on the alumina’s surface is analyzed. We also calculated the PDRC performance of the samples using a radiative transfer model that accounts for the atmospheric conditions and solar irradiance. In addition, it has been studied how the weather conditions affect the cooling performance of the same AAO nanostructure coated with 100 nm of Au, with a special focus on temperatures.

## 2 Materials and Methods

### 2.1 Fabrication of the Nanostructured AAO with Metallic Coatings

To enhance the radiative cooling of AAO nanostructures, they were coated with different metals. The AAO nanostructures were first prepared using a two-step anodization process.^18,28,29^ The anodization was performed in 50 wt % ethylene glycol containing 10 wt % sulfuric acid, at 0 °C, under an applied voltage of 19 V. The first and second anodization times were 24 and 8 h, respectively. Next, an aqueous solution of CuCl_2_ and HCl was used to chemically remove the Al substrate. Then, a 10 wt % H_3_PO_4_ aqueous solution was heated at 30 °C for 10 min to remove the barrier layer. Finally, metallic coatings (Al and Au) with different thicknesses were deposited using an electron beam evaporation system below the AAO nanostructures

### 2.2 Characterization of the Nanostructured AAO with Metallic Coatings

Morphological characterization was conducted using high-resolution field emission scanning electron microscopy (FE-SEM, FEI VERIOS 460) with a 2 kV accelerating voltage. The solar reflectance (*R*) and transmission (*T*) of the AAO nanostructures with the metallic coatings were measured using a UV–vis–NIR PerkinElmer Lambda 950 double beam spectrophotometer equipped with a 150 mm Spectralon-coated integrating sphere. The wavelength range used was from 0.3 to 2.5 μm. Angular specular reflectance spectra were measured by using a PerkinElmer Universal Reflectance Accessory (URA) that allows automatic specular reflectance measurements at different incident angles. The mid-IR reflectance and transmission (from 5 to 17 μm) were measured with a Fourier transform infrared (FT-IR) spectrophotometer from PerkinElmer (Frontier). A 75 mm-diameter integrating gold sphere was incorporated to collect both specular and diffuse reflectance components. Then, the emissivity was obtained by 1 – *R* – *T* for all the wavelengths. The solar irradiance data corresponding with the background AM 1.5 G spectrum was obtained from the National Renewable Energy Laboratory Web site,^30^ and the atmosphere transmission data were available at the Gemini Observatory Web site.^31^

Passive radiative cooling characterization was performed both indoors and outdoors. For the indoor characterization, the tailored indoor setup designed by Song et al.^32^ was used. This setup creates a heat sink using a hemispherical aluminum dome cooled with liquid nitrogen. It is equipped with an air mass (AM) 1.5 solar simulator to measure under conditions analogous to daytime and nighttime field testing. The repeatability has been thoroughly demonstrated for three dissimilar materials: a silver (Ag) mirror, a polydimethylsiloxane (PDMS) film, and a graphite coating. Therefore, prior to the characterization of new samples, the Ag mirror is used for the initial calibration of the setup in such a way that the thermal stabilization of the system is verified by measuring the steady state of the Ag mirror: 23.0 ± 0.8 °C. To ensure a good thermal contact between the AAO nanostructures and the underlying thermocouple, a copper (Cu) bulk was placed on top of the sample holder in the indoor setup. For a more reliable comparison, the Cu bulk was characterized in the absence of a sample: when the solar light is turned on, the Cu bulk has a temperature of 27.0 ± 0.8 °C, and when the solar light is turned off, it remains at 19.5 ± 0.8 °C. The field measurements were carried out on the building’s rooftop using an outdoor characterization setup.^18^ This setup consisted of multiple polystyrene foam blocks covered by Al foil and sealed by using low-density polyethylene (LDPE). K-type thermocouples were in contact with the bottom surface of the coolers to record real-time temperature variations. Multiple cycles of measurement were performed to show 48 h of representative data. One of the thermocouples recorded the temperature in the absence of a cooler, named “empty box”, as a reference of no passive radiative cooler behavior. An Al bulk was also included as a reference. A weather station was placed nearby the setup to collect the weather conditions, namely, solar radiation, air temperature, relative humidity, and wind.

Calculations of cooling power density (*P*_cool_) were carried out using the experimentally obtained emissivity data of every cooler, at the ambient temperature recorded by the weather station, considering a heat-transfer coefficient, *h*_CC_, of 12 W/m^2^·K as representative for the outdoor characterization setup.^18^ The equations^5^ can be found in the Supporting Information (eqs S1–S6).

## 3 Results and Discussion

### 3.1 Effect of the Metal Choice for Coating the AAO Nanostructures

**Figure 1:**
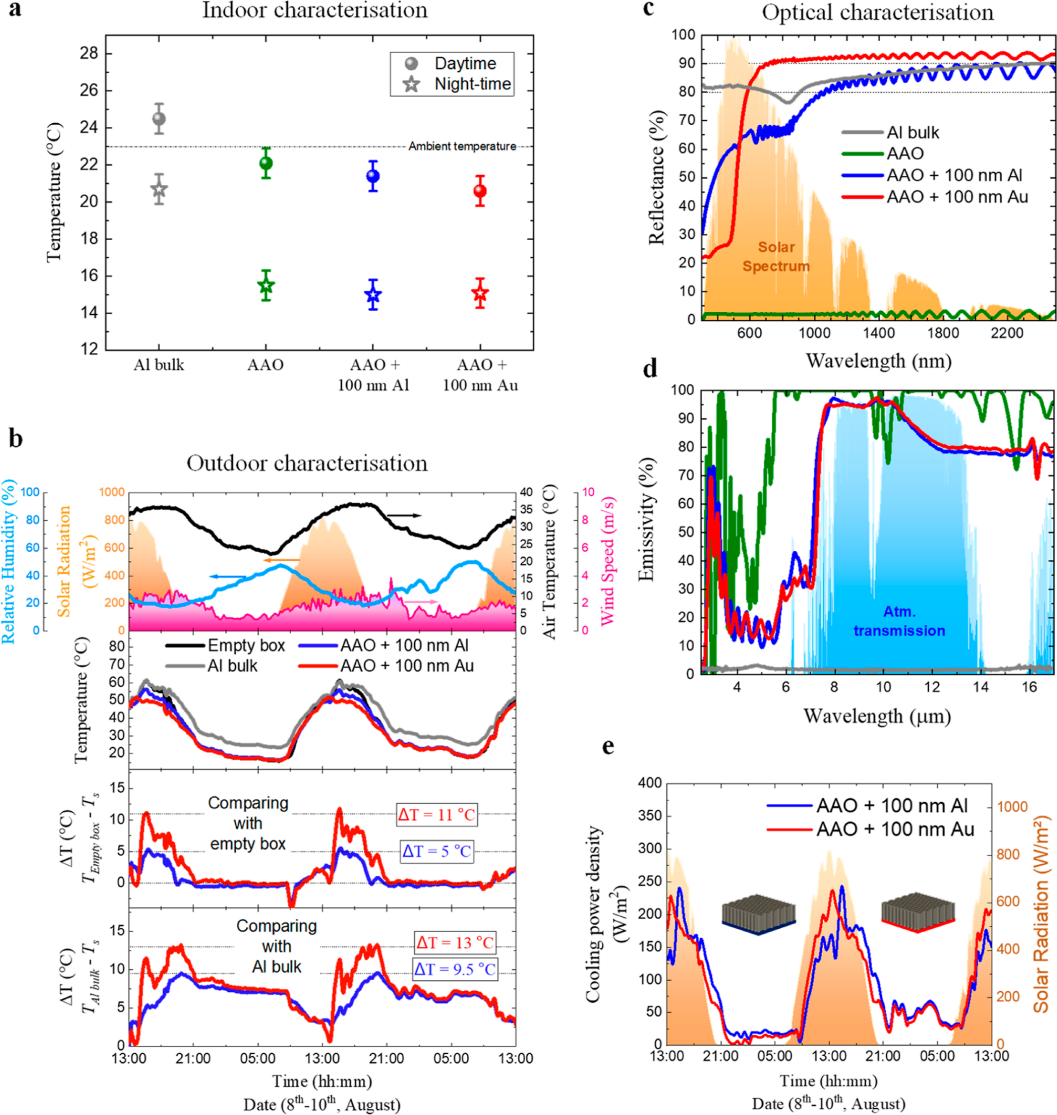
Influence of a metallic coating on the passive radiative cooling performance of the AAO nanostructures. (a) Indoor passive radiative cooling characterization of Al bulk, free-standing AAO nanostructures, and AAO nanostructures coated with 100 nm of Al and Au. Dashed line corresponds to the ambient temperature. (b) Overview of the outdoor passive radiative cooling performance, including the weather conditions, the measured temperatures, and the calculated temperature reductions compared with the empty box and with an Al bulk. The optical characterization includes (c) solar reflectance and (d) IR emissivity spectra. (e) Calculated cooling power density for the AAO nanostructures coated with 100 nm of Al and Au.

To illustrate the influence of the metallic coating, the passive radiative cooling performance of an Al bulk, a free-standing AAO nanostructure, and several free-standing AAO nanostructures coated with 100 nm of Al and Au have been characterized in a tailored indoor setup. The mean temperatures are shown in Figure 1a, when the steady-state temperature observed for the Ag mirror is 23.0 ± 0.8 °C, which is representative of the ambient temperature when there is no passive radiative cooling.

Figure 1a shows that the Al bulk is not able to cool down under daytime conditions, maintaining 24.5 ± 0.8 °C when the solar light is turned on and 20.7 ± 0.8 °C when it is turned off. The free-standing AAO nanostructure shows 22.1 ± 0.8 °C under daytime conditions, in contrast with 15.5 ± 0.8 °C under nighttime conditions. The explanation for these similar daytime results is based on different optical responses: the Al bulk is a good solar reflector (solar reflectance above 80%, see Figure 1c), but the IR emissivity is around 2% (Figure 1d), which is insignificant. Therefore, it becomes moderately hotter by reflecting the solar light and maintains a slightly lower temperature when the solar simulator is turned off. The free-standing AAO nanostructure shows a minimum solar reflectance (around 2%; see Figure 1c), because it is highly transparent for these wavelengths, and also a strong IR emissivity, close to 100% from 6 to 14 μm (see Figure 1d). Both features, the high transparency and the high IR emissivity, allow a temperature reduction (Δ*T*) of 4.9 °C when the solar light is turned on, considering the temperature of the underlying Cu bulk (27.0 ± 0.8 °C) and the temperature of the free-standing AAO nanostructure (22.1 ± 0.8 °C). When the solar light is turned off, the high IR emissivity of the AAO nanostructure allows a Δ*T* of 4.0 °C compared to the Cu bulk temperature (19.5 ± 0.8 °C), while compared to the ambient temperature, Δ*T* is 7.5 °C. However, a material with such low solar reflectance is not suitable for PDRC, despite its strong thermal emission. Hence, to improve the solar reflectance, the free-standing AAO nanostructure has been combined with 100 nm metallic coatings, Al and Au. As can be seen in Figure S1 in the Supporting Information, the transmittance of a free-standing alumina is ∼90% from 300 to 2500 nm. This allows solar radiation to pass through and strike the metallic layer surface directly. A much higher proportion of solar radiation is reflected by the alumina–metal interface due to the high solar reflectances of Al and Au. Therefore, instead of absorbing the radiation and heating, the sunlight is reflected.

The performances of these AAO nanostructures with metallic coatings during the indoor characterization (see Figure 1a) show a similar behavior when the solar light is turned off: 15.0 ± 0.8 and 15.1 ± 0.8 °C for Al and Au coatings, respectively. This result is because both AAO nanostructures have almost identical IR emissivity spectra, as shown in Figure 1d. Neither Al bulk nor Au bulk are thermal emitters; therefore, their IR emissivity is negligible. However, depositing a metallic coating on the AAO nanostructures changes the IR emissivity of the free-standing AAO nanostructure. Interestingly, the deposition of the metal layer on the AAO nanostructures transforms the broadband AAO emitter into a more selective emitter, thereby enhancing the possibility for optimum temperature reductions. When the solar simulator is turned on, the effect of the metallic coating below the AAO nanostructures is more distinct: the AAO nanostructure with 100 nm of Al reaches 21.4 ± 0.8 °C, and the one with 100 nm of Au reaches 20.6 ± 0.8 °C. The temperature reduction achieved by the AAO nanostructure coated with 100 nm of Au is slightly higher than the one reached by the AAO nanostructure coated with 100 nm of Al at room temperature. This difference is due to the higher solar reflectance at wavelengths higher than 700 nm when the AAO nanostructure is coated with 100 nm of Au in contrast to 100 nm of Al.

The cooling ability under daytime conditions strongly depends on both the solar reflectance of the coolers and their temperature. As Figure 1c shows, the AAO nanostructure coated with 100 nm of Au shows a step form in the solar reflectance, being minimum (25%) from 300 to 450 nm and maximum (92%) from 750 to 2500 nm. Then, the one with 100 nm of Al shows a solar reflectance under 70% from 300 to 800 nm and under 90% from 800 to 2500 nm. Because of these differences, the AAO nanostructures with 100 nm of Au and Al have an average solar reflectance (see eq S7 in the Supporting Information) of 75 and 59% for the AAO nanostructures with 100 nm of Au and Al, respectively. The calculated cooling power density for the AAO nanostructures under direct sunlight, at room temperature (300 K), with a *h*_CC_ = 12 W/m^2^·K, results negligible. In contrast, during nighttime, the calculated cooling power density is 123.3 and 123.9 W/m^2^ for 100 nm of Al and 100 nm of Au, respectively. As per eqs S1–S6 in the Supporting Information, the cooling power density increases with the material temperature. Hence, AAO nanostructures become good candidates for high temperatures and outdoor applications. It is noteworthy that the AAO nanostructures are chemically, thermodynamically, and mechanically stable for long-term outdoor applications, even under real outdoor conditions. In contrast, typical polymer-based composites must address possible material degradation when they are used for outdoor applications.^33,34^ Prolonged weather exposure can induce photooxidative degradation on some polymer materials, causing discoloration, loss of gloss, peeling of the surface, and refractive index and mechanical properties reduction.^35−38^ These changes can compromise the passive radiative cooling performance. For this reason, there is extra interest in testing these AAO nanostructures under real weather conditions in warm climates, where it is possible to measure the cooling performance at around 60 °C under direct sunlight conditions.

Figure 1b shows the outdoor passive radiative cooling characterization, performed on the building’s rooftop. It provides an overview of the weather conditions, the measured temperature of the AAO nanostructures together with the two references (empty box and Al bulk), as well as the reduction temperature calculated considering, first, the empty box and, second, the Al bulk. The free-standing AAO nanostructure has been excluded from the outdoor characterization due to its high transparency and low reflectance in the solar spectrum wavelength range because these two features are undesirable for a passive daytime radiative cooler.

The weather conditions are typical for sunny days in Madrid (Spain) in the summer–blue sky, no clouds, no precipitation, and no strong gusts of wind. The air temperature varies between 36 °C (daytime) and 22 °C (nighttime), the relative humidity goes from 50 to 20%, and the solar irradiation reaches maximum values of 820 W/m^2^ around 15:00. Under these weather conditions, the recorded temperature reached by the empty box and the Al bulk shows maxima of over 61 °C under direct sunlight. During the nighttime, the Al bulk maintains a temperature 7 °C higher than the empty box. The variations in the AAO nanostructures’ temperature are better described in terms of temperature reduction. In comparison with the empty box, during the daytime, the temperature of the AAO nanostructure coated with 100 nm of Au is reduced by 11 °C, while the temperature of the AAO nanostructures coated with 100 nm of Al shows a maximum reduction of 5.3 °C. When compared to the Al bulk, the peaks in the temperature reduction are 13 °C for the AAO nanostructure coated with 100 nm of Au and 9.5 °C for the one coated with 100 nm of Al. Several peaks are reached later at 19:00. We attribute this to the low IR emissivity of the Al bulk, which does not allow radiative heat transfer. Therefore, the temperature of the Al bulk decreases exclusively because of nonradiative exchanges of heat. These processes are slower and less effective than passive radiative cooling due to the design of the outdoor setup, as the AAO nanostructures illustrate. During the nighttime, the temperature of both AAO nanostructures remains 7 °C below the Al bulk’s temperature.

The cooling power density of both AAO nanostructures with coatings can be calculated considering the conditions measured during outdoor characterization. This includes the actual temperature measurements of each nanostructure, the measured ambient temperature, and recorded solar radiation data. These calculations provide a visual representation of the performance differences between the AAO nanostructures due to the metallic coating.

As depicted in Figure 1d, the cooling power density of both AAO nanostructures stabilizes at minimum values between 15 and 50 W/m^2^ during nighttime. However, notable differences in the cooling power density are observed during daytime. From sunrise at 6:30 until 8:45 a.m., when solar radiation is 330 W/m^2^, the estimated cooling power density is around 10 W/m^2^ for both AAO nanostructures. As solar radiation increases, the differences in the cooling power density also increase. At 11:30 am, with a solar radiation of 724 W/m^2^, the AAO nanostructure coated with 100 nm of Au achieves a cooling power density of 170 W/m^2^, compared to the 130 W/m^2^ achieved by the AAO nanostructure coated with 100 nm of Al.

The maximum difference occurs at 13:15, when the cooling power density is 238 W/m^2^ for the AAO nanostructures coated with 100 nm of Au and 169 W/m^2^ for the one coated with 100 nm of Al. This difference in the estimated cooling power density can be attributed to the average solar reflectance of the AAO nanostructures. When the coating is 100 nm of Au, the average solar reflectance is 15% higher than when it is 100 nm of Al.

As a result, the maximum temperatures reached by the AAO nanostructure coated with 100 nm of Au are around 50 °C between 13:15 and 16:00, while the maximum temperatures reached by the AAO nanostructure coated with 100 nm of Al peak at 56 °C at 15:00. However, when this nanostructure reaches 56 °C, its cooling power density increases to 243 W/m^2^, enhancing cooling from 15:00 to 18:00.

Therefore, although the maximum cooling power density values are similar, they occur 2 h apart. The AAO nanostructure coated with 100 nm of Au reaches its maximum cooling power density earlier and achieves a higher temperature reduction under direct sunlight.

### 3.2 Effect of the Metal Coating Thickness on the AAO Nanostructures

The characteristics of the AAO nanostructures with metallic coatings are a combination of the free-standing AAO nanostructures’ properties with the metal’s properties, as has been extensively discussed in the previous section. For this reason, the thickness of the metallic coatings is expected to be an important parameter.

To study the influence of the metal coating thickness on the AAO nanostructures, multiple cases have been analyzed, namely, 20, 40, 60, and 100 nm for both Al and Au. These thicknesses have been chosen to include from an initial case where the metallic layer is too thin to cover completely the AAO nanostructure’s surface to a thick enough layer, whose properties are like those of the metal bulk.

**Figure 2:**
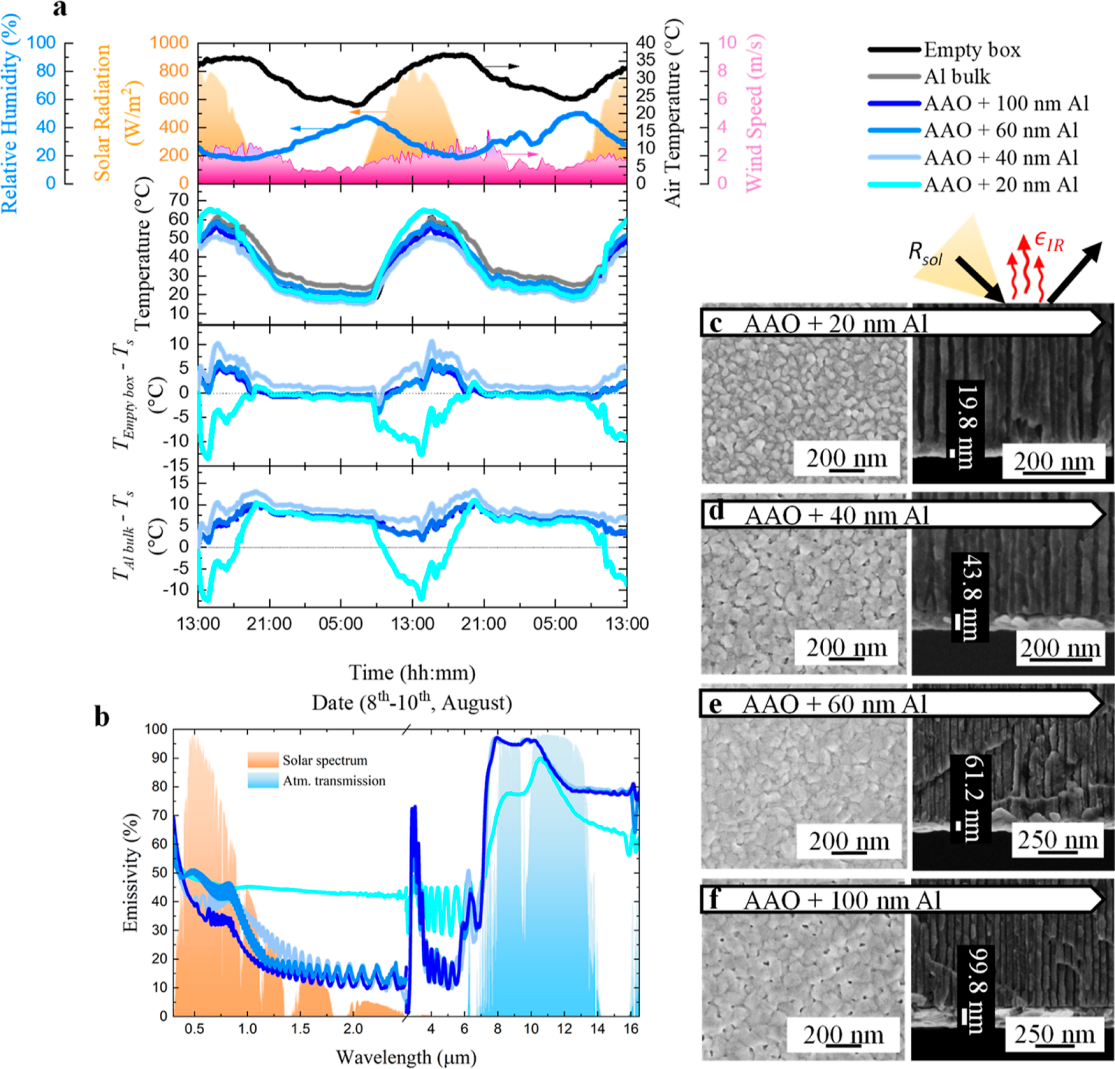
Analysis of the passive radiative cooling performance of the AAO nanostructures coated with different thicknesses of Al. (a) Overview of the outdoor passive radiative cooling performance, including the weather conditions, the measured temperatures, and the calculated temperature reductions and comparison with the empty box and with an Al bulk. (b) Emissivity spectra and (c–f) FE-SEM images of the bottom view and cross-section of the AAO nanostructures to show the Al layers, surface, and measured thickness for the nominal values of 20, 40, 60, and 100 nm.

All the AAO nanostructures coated with Al have been characterized simultaneously to discuss the differences in their passive radiative cooling performances outdoor, under real weather conditions. Figure 2a provides an overview of the weather conditions and the measured temperature of the AAO nanostructures together with both references (empty box and Al bulk) as the reduction temperature calculated considering, first, the empty box and second, the Al bulk.

The weather conditions are typical for sunny days in Madrid (Spain) in the summer–blue sky, no clouds, no precipitation, and no strong gusts of wind. The air temperature varies between 36 °C (daytime) and 22 °C (nighttime), the relative humidity goes from 50 to 20%, and the solar irradiation reaches maximum values of 820 W/m^2^ around 15:00.

Under these daytime weather conditions, the AAO nanostructure coated with 100 nm of Al reached a maximum temperature of 56 °C, which corresponds to a temperature reduction of 5.3 °C compared to the empty box. When the Al thickness is reduced to 60 nm, the changes in the cooling performance of the AAO nanostructure are tiny; the maximum temperature reduction is 5.5 °C. However, by reducing the Al thickness to 40 nm, the AAO nanostructure reached a maximum temperature reduction of 10.5 °C. In contrast, the AAO nanostructure coated with only 20 nm of Al does not cool but heats up; it gets 12 °C warmer than the empty box under direct sunlight. The distinct responses are linked to the emissivity of every AAO nanostructure, as can be seen in Figure 2b. High solar emissivity means high solar absorptivity; therefore, the AAO nanostructure coated with 20 nm of Al with an average solar emissivity of 46% absorbs a massive amount of solar radiation. This explains why this AAO nanostructure heats up. When compared to the other Al thicknesses, the average solar emissivity is 31, 41, and 35% for 100, 60, and 40 nm of Al. In these cases, further explanations are needed to understand the differences in the passive radiative cooling performance. The morphology of the AAO nanostructures is extraordinarily reproducible due to the fabrication process; the top-view and the cross-sectional FE-SEM images are shown in Figure S2 in the Supporting Information. Therefore, it is important to focus on the morphology of the Al layers. As Figure 2c–f shows, there is a progression in the Al layer’s morphology related to the deposited thickness. For 20 nm, small grains are observed forming agglomerates, which do not cover the AAO nanostructure’s surface completely. For 40 nm, the grains are coalescing, but the surface is not yet covered, and there are air pores embedded in the Al layer. For 60 nm, the grains have coalesced, covering the AAO nanostructure’s surface completely. In addition, for 100 nm, the layer that was already completely covered shows surface defects as small holes. It is important to take into account that the different observed defects of the studied metals in the SEM images do not have any effect on the optical properties or cooling performance of the AAO nanostructures coated with Al. Therefore, for passive radiative cooling under direct sunlight, an optimum thickness of 40 nm appeared for the AAO nanostructures coated with Al because the metallic layer is porous and covers the entire AAO surface.

During nighttime, the AAO nanostructures coated with Al show a steady-state temperature like the empty box. The AAO nanostructure coated with 40 nm of Al is the only one that has a small passive radiative cooling effect, keeping the temperature down by 1.4 °C. It is important to note that the temperature reduction is significantly lower than that during the daytime because of the lower ambient temperatures during the night, around 25 °C. This limits the cooling power density of the AAO nanostructures, and therefore, it reduces the passive radiative cooling effect.

Nevertheless, the passive radiative cooling performance of the AAO nanostructures contributes to increasing thermal comfort inside the building during both daytime and nighttime. The performance of the AAO nanostructures with 40 nm of Al stands out among the others: at daytime, it reduces the rooftop temperature from 61 to 50.5 °C, and at night, it reduces the rooftop temperature from 25 to 23.6 °C.

For further verification of the effect of the Al thickness on the AAO nanostructures, the temperature reductions have been compared with the Al bulk. Here, the peaks in the temperature reduction are reached later, at 19:00. The reason for this is the extremely low emissivity of the Al bulk. However, the AAO nanostructures coated with 100, 60, and 40 nm of Al can cool to 3.3, 3.5, and 6.5 °C, respectively, when the solar radiation is at its maximum at 15:00. Then, the temperature reduction becomes higher, up to maximum reductions of 9.6, 10.7, and 13.2 °C for the AAO nanostructures coated with 100, 60, and 40 nm of Al. This trend observed for passive radiative cooling compared with the Al bulk is analogous to that observed compared to the empty box. In contrast again, the AAO nanostructure with 20 nm of Al is heated 12 °C above the Al bulk temperature, corroborating that the 20 nm of Al thickness is too thin to reflect solar radiation efficiently.

During nighttime, the steady-state temperature of the nanostructures is 7 °C below the Al bulk temperature for the AAO nanostructures coated with 100 and 60 nm of Al, 8 °C for the AAO nanostructure coated with 40 nm of porous Al layer, and 6 °C for the AAO nanostructure coated with 20 nm of Al. This last temperature reduction is the lowest because of the differences in the IR emissivity values for wavelengths between 8 and 13 μm (see Figure 2b) at the atmospheric window.

In conclusion, regarding all the details about the different passive radiative cooling performances of the AAO nanostructures, the one coated with 40 nm of Al reaches a maximum temperature reduction 2.5 °C higher than the previous published work^18^ under similar weather conditions and under direct sunlight. This improvement is achieved by reducing the Al thickness from a bulk material (500 μm) to 40 nm.

**Figure 3:**
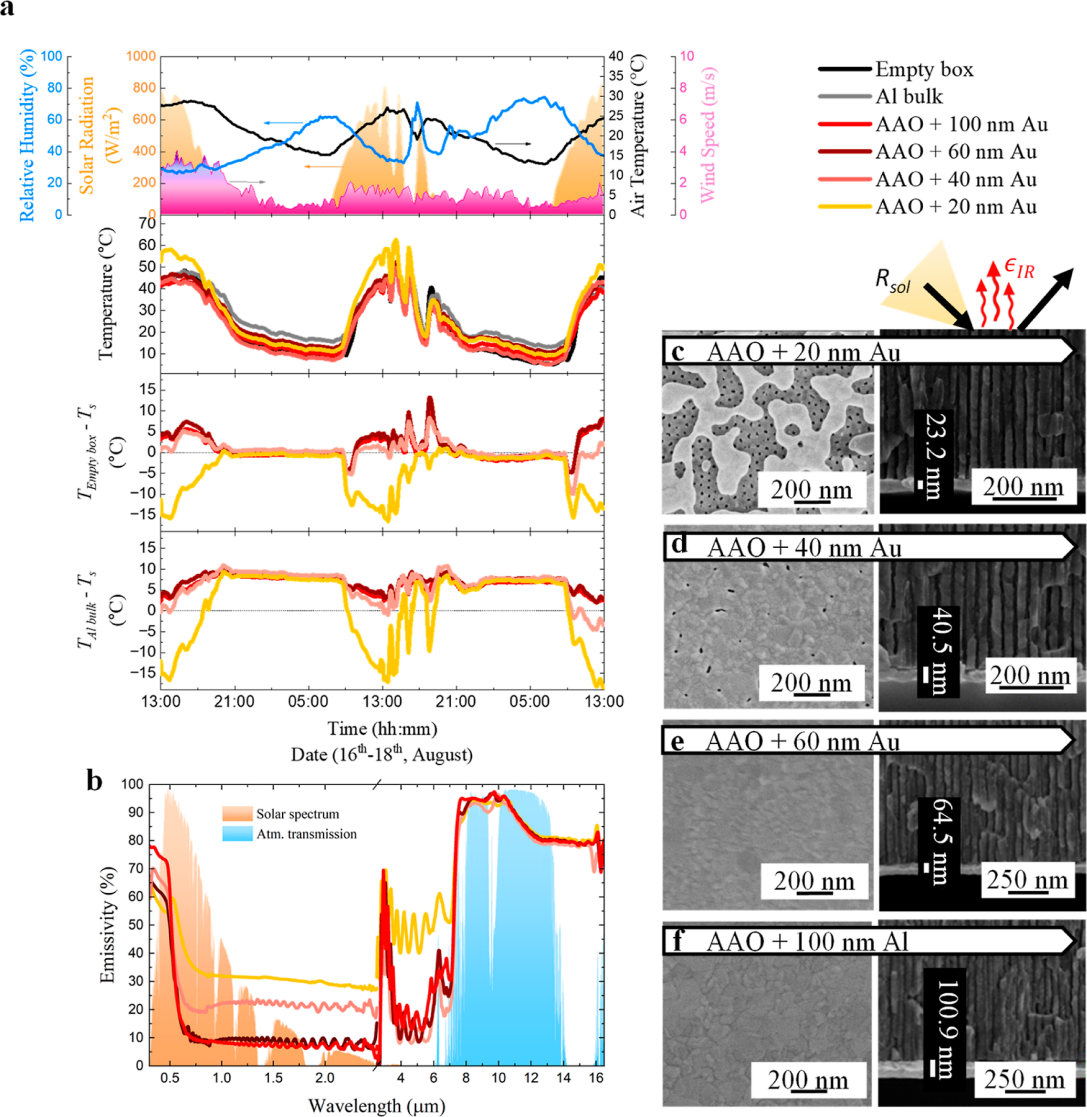
Analysis of the passive radiative cooling performance of the AAO nanostructures coated with different thicknesses of Au. (a) Overview of the outdoor passive radiative cooling performance, including the weather conditions, the measured temperatures, and the calculated temperature reductions and comparison with the empty box and with an Al bulk. (b) Emissivity spectra and (c–f) FE-SEM images of the bottom view and cross-section of the AAO nanostructures to show the Au surface and measured thickness for the nominal values of 20, 40, 60, and 100 nm.

Despite the great passive radiative cooling performance shown by the AAO nanostructures coated with Al, the results obtained during the indoor characterization suggest that the AAO nanostructures coated with Au could show even better performance under real weather conditions. Therefore, all the AAO nanostructures coated with different thicknesses of Au have been characterized simultaneously to discuss the differences in their passive radiative cooling performances outdoor. Figure 3a provides an overview of the weather conditions, the measured temperature of the AAO nanostructures together with the two references (empty box and Al bulk), and the reduction temperature calculated considering, first, the empty box and, second, the Al bulk.

The weather conditions correspond to a summer day in Madrid (Spain), including a smooth summer storm during the cycle of measuring. The air temperature varies between 29 °C (daytime) and 15 °C (nighttime), the relative humidity goes from 64 to 25%, the maximum solar irradiation is ≈800 W/m^2^, and there are no strong gusts of wind. Under these daytime weather conditions, the maximum temperature recorded in the empty box is 48 °C. In comparison, the temperature of the AAO nanostructure coated with 20 nm of Au is 15 °C higher than the empty box temperature, while the AAO nanostructures coated with 40, 60, and 100 nm of Au show a maximum temperature reduction of 7.5, 12.5, and 10.4 °C, respectively, under direct sunlight. The relationship between the reduction temperature and the Au thickness agrees with the variations in the solar emissivity spectra, as can be seen in Figure 3b. Figure 3b shows that the AAO nanostructure coated with 20 nm of Au has the lowest  (60%) at wavelengths below 550 nm but the highest  at longer wavelengths (30%). The AAO nanostructures coated with 60 and 100 nm of Au show stronger differences in these  regions, 75 and 80% at wavelengths below 550 nm, respectively. Then, both  spectra decrease until 8%, and this value is maintained for wavelengths between 700 and 2500 nm. By comparison, the AAO nanostructures with 40 nm of Au show an emissivity spectrum in between, reaching 65% for wavelengths below 550 nm and  of 20% from 700 to 2500 nm. Thus, the average solar emissivity of the AAO nanostructures is 42, 32, 23, and 25% for 20, 40, 60, and 100 nm, respectively. Therefore, the solar reflectivity provided by the AAO nanostructure with 20 nm of Au is not enough to manage absorbed solar radiation. It improves with the Au thickness up to the optimum value of 60 nm (ε_sol_ = 23%), where the solar emissivity is the minimum, and for thicker Au layers, it tends to stabilize without showing a significant improvement for passive cooling (ε_sol_ = 25%).

Analogous to the Al discussion above, the morphology of the AAO nanostructures is extraordinarily reproducible (top-view and cross-sectional FE-SEM images are shown in Figure S2 in the Supporting Information), and further understanding of the differences between the passive cooling performances of the AAO nanostructures coated with Au can be achieved by the morphological characterization of the Au layers.

Figure 3c–f shows the FE-SEM images of the Au surface over the AAO nanostructure (bottom view of the samples) together with the cross-section. A clear evolution can be observed with an increasing Au thickness. For 20 nm, there are Au islands that maintain the AAO nanostructure surface uncovered. For 40 nm, the AAO nanostructure surface is completely coated by the grainy layer of Au. For 60 nm, the grains coalesced, covering the AAO nanostructure surface completely, and for 100 nm, there are minor changes related to the grain size that has been enlarged. However, once the AAO nanostructure surface has been completely coated by the Au layer, there is no further enhancement in the passive radiative cooling capability. Therefore, the AAO nanostructure coated with Au shows an optimum metallic thickness of 60 nm under direct sunlight.

During the night, the temperature on the rooftop is around 12 °C as measured in the empty box. At this temperature, the steady state of the AAO nanostructure is quite close to the empty box without further cooling due to the limited cooling power density at low temperatures. Nevertheless, the presence of the AAO nanostructures coated with Au contributes to a more efficient temperature management during both daytime and nighttime, as discussed in the AAO nanostructures coated with Al. The performance of the AAO nanostructures with 60 nm of Au stands out among the others: at daytime, it reduces the rooftop temperature from 48 to 35.5 °C, and at night, it maintains at 12 °C.

For further analysis of the changes in the metallic coating, the temperature reduction achieved by the AAO nanostructures coated with Au has also been compared with that of an Al bulk. Here, analogously to the AAO nanostructure coated with Al, the peaks in the temperature reduction are reached later, at 19:00, due to the low emissivity of the Al bulk. During the daytime, the AAO nanostructures with 100, 60, and 40 nm of Au can cool to 2.5, 3, and 0 °C, respectively, when the solar radiation is at its maximum at 15:00. Then the temperature reduction compared with the Al bulk increases up to 7.8, 8.5, and 7.8 °C for 100, 60, and 40 nm of Au, respectively. These temperature reductions are maintained during the nighttime. These values are close to each other because there is no significant difference in the IR emissivity spectra of the AAO nanostructures coated with Au (see Figure 3b). The most extreme behavior is described by the temperature of the AAO nanostructure coated with 20 nm of Au. In comparison to the Al bulk, this nanostructure heats up to 16 °C under direct sunlight, and it shows a steady-state temperature reduction of 7.8 °C during nighttime. This corroborates that 20 nm of Au is too thin to reflect solar radiation efficiently. Moreover, the passive radiative cooling performance is improved by a thicker Au layer up to achieve an optimum thickness of 60 nm of Au.

In conclusion, regarding all of the details of the multiple passive radiative cooling performances of the AAO nanostructures, the one coated with 60 nm of Au reaches a maximum temperature reduction of 2 °C higher than the AAO nanostructure with 40 nm of Al. This improvement also means a temperature reduction of 4.5 °C higher than that reported in previous work^18^ with AAO nanostructures under similar weather conditions. In addition, this result provides an alternative way to boost the cooling performance of distinct radiative coolers.

### 3.3 Sensitivity of AAO Nanostructures’ Cooling Power to Weather Variations

Additionally, a comparative study of the passive radiative cooling performance has been carried out over several weeks to show the passive modulation of the cooling power density of the AAO nanostructures due to weather variations. To study this effect, as a first approximation, several calculations of the *P*_cool_ have been carried out, including representative daytime and nighttime conditions for each day. It is worth noting that variations in relative humidity, atmospheric transmission, or solar radiation have not been included for simplicity in the calculations. Therefore, the changes in *P*_cool_ might be underestimated.

**Figure 4:**
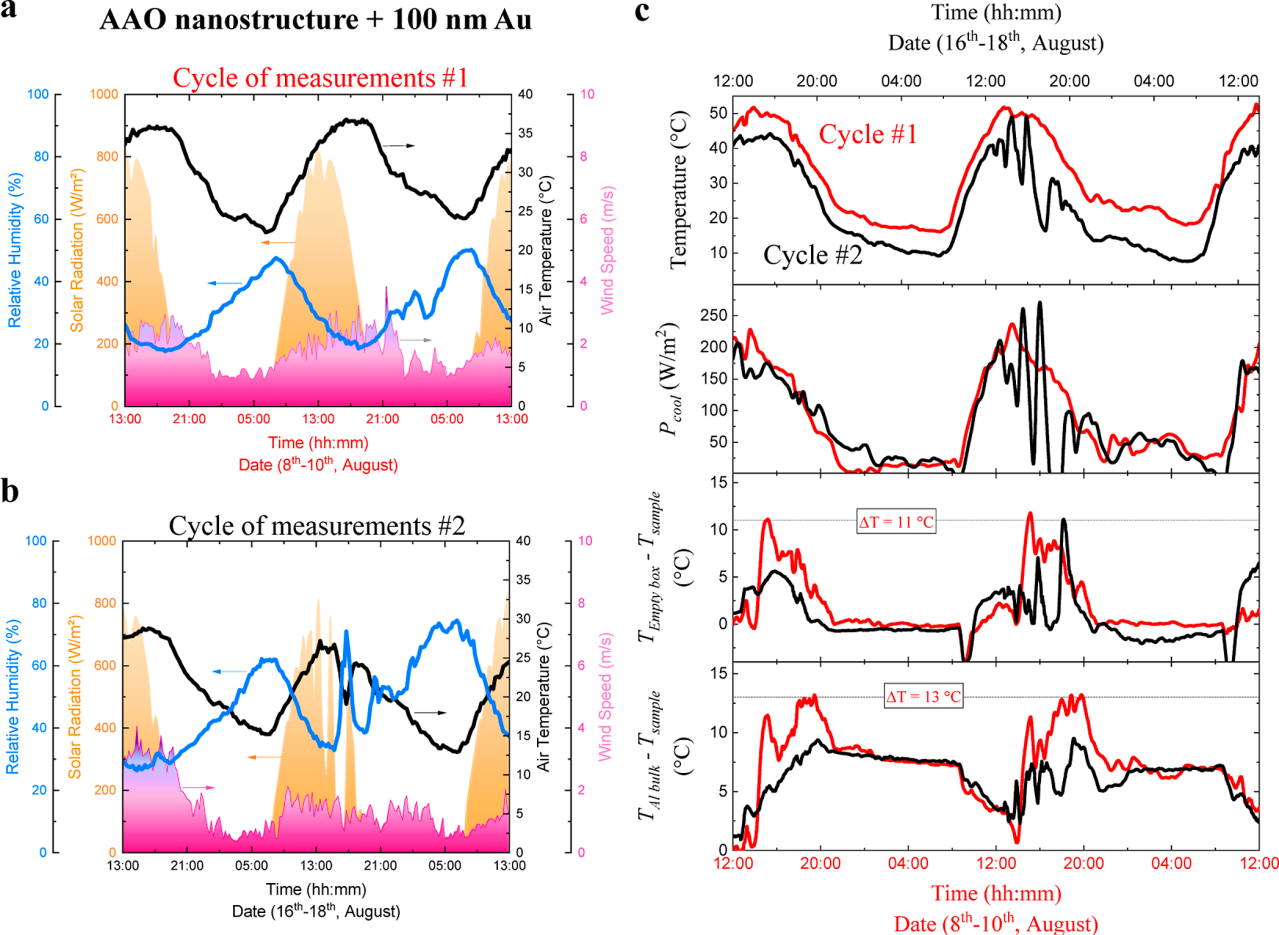
Variations on the passive radiative cooling performance of the AAO nanostructures coated with 100 nm of Au due to the change in weather conditions. Weather conditions during (a) cycle #1 and (b) cycle #2. (c) Overview of the analysis of the cooling performance considering the measured temperatures, the calculated cooling power density, and the obtained temperature reduction and comparison with the empty box as well as with an Al bulk.

Figure 4 shows the details about the weather conditions corresponding to cycles 1 and 2, together with an overview of the cooling performance of the AAO nanostructure coated with 100 nm of Au during both cycles. The overview (Figure 4c) includes the measured temperature, the calculated cooling power density, and the temperature reduction compared, first, with the empty box and, second, with the Al bulk.

The weather during cycle #1 was sunny, without clouds. The sky was blue, and there was neither precipitation nor strong gusts of wind. The ambient temperature was between 36 °C (daytime) and 22 °C (nighttime), the relative humidity went from 50 to 20%, and the solar irradiation reached maximum values of 820 W/m^2^ around 15:00, as can be seen in Figure 4a. The maximum temperature recorded on the rooftop was 61.3 °C under direct sunlight. However, the weather during cycle #2 included a short, smooth summer storm, although it was mostly sunny and without strong gusts of wind too. The ambient temperature varied between 29 °C (daytime) and 15 °C (nighttime), the relative humidity went from 64 to 25%, and the maximum solar irradiation was ≈800 W/m^2^ at 15:00 (see Figure 4b). The maximum temperature recorded on the rooftop was 48 °C. Therefore, cycle #1 was hotter and sunnier than cycle #2, while cycle #2 showed a higher relative humidity and less ambient temperature than cycle #1.

In Figure 4c, the red lines refer to cycle #1 and the black lines refer to cycle #2, and all of the lines correspond to the AAO nanostructure coated with 100 nm of Au. As can be seen, the maximum temperatures measured during cycle #1 are between 51 and 52 °C, and the minimum temperatures recorded are between 17 and 18 °C. In contrast, during cycle #2, the maximum temperatures are between 43 and 49 °C, and the minimum temperatures are between 11 and 9 °C. Hence, the AAO nanostructure coated with 100 nm of Au is at lower temperatures during cycle #2 than during cycle #1 because of the weather variations. The AAO nanostructure temperature is closely related to its instantaneous cooling power. The calculated cooling powers are similar during nighttime because of the low temperatures of the AAO nanostructure. In contrast, during the day, there are differences in the maximum cooling power density, which reaches 230 W/m^2^ during cycle #1 and is around 170–200 W/m^2^ during cycle #2. It is important to note that before the summer storm, the AAO nanostructure’s temperature is naturally lower (around 5 °C), and after the summer storm, the temperature decreases even more (around 10 °C). The peaks obtained during the summer storm due to the sudden drop in temperature are deliberately ignored. Therefore, the calculated power density for the AAO nanostructure coated with 100 nm of Au is lower during cycle #2 than during cycle #1. Accordingly, during the daytime, the temperature reduction compared with the empty box is between 11.8 and 11.1 °C during cycle #1 and between 5.6 and 7 °C during cycle #1. Furthermore, by comparing with the Al bulk, the temperature reduction under direct sunlight goes from 1 to 13 °C in cycle #1 and from 3 to 9.4 °C in cycle #2. The thermal contrast is more marked in cycle #1, while cycle #2 has a milder weather condition, and in both cycles, the AAO nanostructure passively contributes to cooling down the temperature to achieve higher comfort by reducing thermal stress. The fact that the AAO nanostructures do not cool down at room temperature would allow a cooling system based on this material to remain exposed all day.

It is remarkable that none of the AAO nanostructures coated with metals used in this work show any signs of deterioration, damage, or degradation associated with outdoor characterization. There is no evidence of weather-related changes due to UV exposure or rain. However, if necessary for high-humidity environments, the surface of the nanostructures could be treated with stearic acid (STA) to increase their hydrophobicity.^39^

Anodization of Al is a process normally used to prevent oxidation of the Al component, and it can be carried out on a large scale. Therefore, it could be implemented in different parts of buildings such as windows, terraces, or roofed verandas in order to enhance the heat management. In addition, the metal coating process is performed using an electron beam evaporation system. This system is commonly used in industry for depositing large areas. Hence, both approaches, Al anodizing and metal coating, are perfectly used as industrial processes, even for large surfaces. Manufacturing costs of these AAO nanostructures coated with 100 nm of metal have been estimated using the production price reported in ref (40) for the AAO nanostructures and the average price of the metal. According to ref (40), great results can be achieved by fabricating AAO nanostructures using low-purity Al at an estimated cost of 0.008 €/cm^2^. Then, the relative cost of adding 100 nm of Au increases the price up to 0.0216, 8.01 × 10^–3^, and 8.00 × 10^–3^ €/cm^2^, respectively.

## 4 Conclusions

In this study, we demonstrated that depositing a metallic layer on the surface of anodic alumina oxide nanostructures significantly enhances their passive radiative cooling performance. Our investigation focused on two metals: Au and Al, with varying thicknesses (20, 40, 60, and 100 nm). It has been found that the optimum Al metallic thickness is 40 nm and that for Au is 60 nm. These combinations provide the highest cooling performance: a maximum temperature reduction of 10.5 °C for the AAO nanostructures coated with 40 nm of Al and 12.5 °C for the AAO nanostructure coated with 60 nm of Au.

The sensitivity of the AAO nanostructures to weather variations has also been studied through outdoor passive radiative cooling characterization. Remarkably, these nanostructures hold great promise for thermal management applications at high temperatures, where other materials such as polymer-based composites fail. During the daytime, when cooling is essential, the AAO nanostructures coated with metals reach a good passive radiative cooling performance. Actually, the AAO nanostructures coated with 60 nm of Au passively reduce the temperature by 12.5 °C. Importantly, during the nighttime (when the ambient temperature is already more comfortable), they maintain the ambient temperature. Consequently, these passive cooling nanostructures can significantly enhance thermal comfort, mitigate solar heating, and reduce the need for energy-intensive air conditioning systems in buildings.

Our findings pave the way for innovative solutions in elevated temperature applications, where the thermal stress causes degradation and failure of other types of materials. By harnessing the potential of AAO nanostructures and strategic metal coatings, we can create more comfortable and environmentally conscious spaces.

## Data Availability

All data are available in the main text and/or the Supporting Information.

## Supporting Information Available

Calculations of cooling power density, average solar reflectance, transmittance of a free-standing AAO nanostructure, and FE SEM images of the free-standing AAO nanostructures (PDF)
