# Supplementary material for: Metallic Coatings Boost the Cooling Power of Nanoporous Alumina
Source: ACS Appl Eng Mater. 2024 Jul 29;2(8):2069–79. doi: 10.1021/acsaenm.4c00245 (PMC11348419; doi:10.1021/acsaenm.4c00245)
Supplement: Supplementary file 1 — em4c00245_si_001.pdf [file em4c00245_si_001.pdf]

Supporting Information

Metallic coatings boost the cooling power of nanoporous alumina

Alba Díaz-Lobo[a], Marisol Martin-Gonzalez[a,*], Qimeng Song[b], Ángel Morales-Sabio[c], Markus Retsch[b], Cristina V. Manzano[a]

[a]*Instituto de Micro y Nanotecnología, IMN-CNM, CSIC (CEI UAM+CSIC), Isaac Newton, 8, E-28706, Tres Cantos, Madrid, Spain*

[b]*Department of Chemistry, Physical Chemistry I, University of Bayreuth, 95447 Bayreuth, Germany*

[c]*Centro de Investigaciones Energéticas, Medioambientales y Tecnológicas (CIEMAT), Avda. Complutense, 22, E-28040, Madrid, Spain*

Calculations of cooling power density

The density of cooling power, P_{cool}, has been calculated based on the emissivity measurements from UV-Vis-NIR to MIR range. The following contributions have been considered,

$$P_{cool}(T) = P_{rad}(T) - P_{atm}(T_{amb}) - P_{sun} - P_{cc}(T, T_{amb}) \quad \text{(S1)}$$

where T is the temperature of the coolers, $P_{rad}(T)$ is the density of power radiated out, $P_{atm}(T_{amb})$ is the density of absorbed atmospheric thermal radiation, P_{sun} is the density of absorbed solar radiation, and $P_{cc}(T, T_{amb})$ is the density of power that includes the parasitic loss due to non-radiative exchanges (conduction and convection).

$P_{rad}(T)$ is defined as:

$$P_{rad}(T) = \iint_0^\infty I_{BB}(T,\lambda)\epsilon(\lambda,\theta)\, d\lambda \cos\theta \; d\Omega \quad \text{(S2)}$$

where λ is the wavelength, θ the polar angle and $\int d\Omega$ the angular integral over a hemisphere. $I_{BB}(T,\lambda)$ is the spectral radiance density of a blackbody, which is defined as:

$$I_{BB}(T,\lambda) = \frac{2hc^2}{\lambda^5}\frac{1}{e^{hc/\lambda k_b T} - 1} \quad \text{(S3)}$$

where h is the Planck's constant, c is the speed of light, and k_b is the Boltzmann's constant.

$P_{atm}(T_{amb})$ is defined as:

$$P_{atm}(T_{amb}) = \iint_0^{\infty} I_{BB}(T_{amb},\lambda)\epsilon(\lambda,\theta)\epsilon_{atm}(\lambda,\theta)\, d\lambda \cos\theta \; d\Omega \quad (S4)$$

where $\epsilon_{atm}(\lambda,\theta) = 1 - t(\lambda)^{1/\cos\theta}$ describe the atmosphere's emissivity, and $t(\lambda)$ is the atmosphere's transmittance in the zenith direction.

P_{sun} is defined as:

$$P_{sun} = \int \epsilon(\lambda,\theta) I_{AM1.5}(\lambda)\, d\lambda \quad (S5)$$

where $I_{AM1.5}$ is the standard AM1.5G spectrum of the solar irradiance. The incidence angle of the solar irradiance (θ_{sun}) is fixed, therefore there is no an angular integral in this term, but cos (θ_{sun}) factor.

$P_{cc}(T, T_{amb})$ is defined as:

$$P_{cc}(T, T_{amb}) = h_{cc}(T_{amb} - T) \quad (S6)$$

where h_{cc} is the heat-transfer coefficient.

Average solar reflectance

The average solar reflectance, $\overline{R_{sol}}$, is defined as:

$$\overline{R_{sol}} = \frac{\int_{0.3\,\mu m}^{2.5\,\mu m} R_{sol}(\lambda,\theta) I_{AM1.5}(\lambda)\; d\lambda}{\int_{0.3\,\mu m}^{2.5\,\mu m} I_{AM1.5}(\lambda)\; d\lambda} \quad (S7)$$

where R_{sol} is the solar reflectance, $I_{AM1.5}$ is the standard AM1.5G spectrum of the solar irradiance, λ is the wavelength, and θ the polar angle.

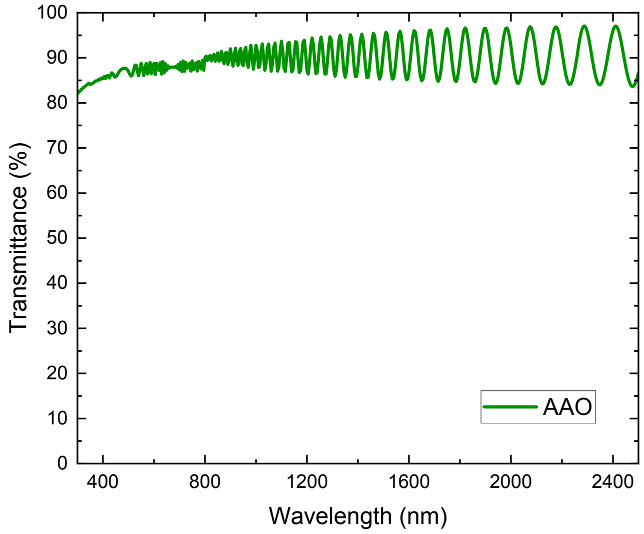

Figure S1: Transmittance of a free-standing AAO nanostructure, without metal coating.

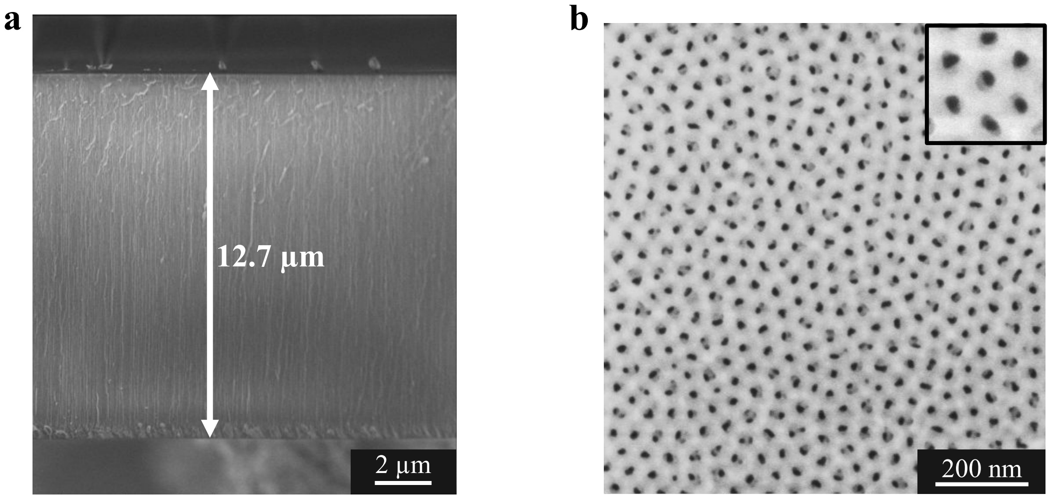

Figure S2. FE-SEM images of the free standing AAO nanostructures: (a) cross-section and (b) top view, the insight shows the hexagonal arrangement of the pores.

Author information

Corresponding author:

*Email: marisol.martin@csic.es
